# A new species of *Harpactea* Bristowe, 1939 from Turkey (Araneae: Dysderidae)

**DOI:** 10.3897/BDJ.3.e4419

**Published:** 2015-09-07

**Authors:** Recep Sulhi Özkütük, Mert Elverici, Yuri M. Marusik, Kadir Boğaç Kunt

**Affiliations:** ‡ Anadolu University, Science Faculty, Biology Department, Eskişehir, Turkey; § Middle East Technical University, Biology Department, Ankara, Turkey; | Erzincan University, Science and Arts Faculty, Biology Department, Erzincan, Turkey; ¶ IBPN RAS, Magadan, Russia

**Keywords:** Alanya, Antalya, Mediterranean, spider, woodlouse hunters

## Abstract

A new species of *Harpactea* Bristowe, 1939, *H. alanyana* **sp. n.** is described from southern Turkey. The new species appears closely related to *H. osellai* Brignoli, 1978. Detailed description and illustrations of the new and related species are provided. The relationships of the two species are discussed.

## Introduction

*Harpactea* Bristowe, 1939 is large genus of dysderid spiders that includes 172 species distributed in the Mediterranean region from the Iberian Peninsula to Turkmenistan (World Spider Catalog 2014). In spite of this wide distribution range and high species richness, comments in the literature highlighted that the genus has been inadequately studied and there is an urgent need for extensive revisionary studies (Alicata 1966b, Alicata 1966a, Chatzaki and Arnedo 2006). First record of *Harpactea* in Turkey has been given by Nosek (1905) from Istanbul [*H. babori* (Nosek, 1905)], whilst most of the known species were described by Brignoli from Mediterranean and Blacksea coasts of Turkey (Brignoli 1978a, Brignoli 1978b, Brignoli 1979). Most of the Turkish species are local endemics, known only from their type localities, but sometimes also from proximate localities. The genus is currently represented by 23 species in Turkey (Bayram et al. 2014).

The goal of this article is to describe a recently discovered species of *Harpactea* from Turkey on the basis of both sexes.

## Materials and methods

Specimens were collected from Antalya Province in the Mediterranean region of Turkey, using a sifter. The specimens were preserved in 70% ethanol and deposited in the Anadolu University Zoology Museum. Digital images of the copulatory organs were taken with a Leica DFC295 digital camera attached to a Leica S8AP0 stereomicroscope and 5-15 photographs were taken in different focal planes and combined using automontage software. SEM microphotographs were made from dried and sputter coated (by gold) organs by use of a Zeiss Ultra Plus SEM device (Anadolu University, Eskişehir). All measurements are in mm, with methods as per (Chatzaki and Arnedo 2006). Terminology for the copulatory organs is adapted from (Alicata 1966a) and (Deeleman-Reinhold 1993).

### Abbreviations

The following abbreviations are used in the text: ***Carapace and abdomen*: AL**, abdominal length; **CL**, carapace length; **CWmax**, maximum carapace width; *CWmin*, minimum carapace width. ***Eyes*: AME**, anterior median eyes; **PLE**, posterior lateral eyes; **PME**, posterior median eyes; **AMEd**, diameter of anterior median eyes; **PLEd**, diameter of posterior lateral eyes; **PMEd**, diameter of posterior median eyes. ***Chelicera*: ChF**, length of cheliceral fang; **ChG**, length of cheliceral groove; **ChL**, total length of chelicera (lateral external view). ***Legs*: Ta**, tarsus; **Me**, metatarsus, **Ti**, tibia; **Pa**, patella; **Fe**, femur; **Tr**, trochanter; **C**, coxa; **D**, dorsal; **Pl**, prolateral; **Rl**, retrolateral; **V**, ventral.

### Depository

**AUZM**, Anadolu University Zoology Museum, Eskişehir, Turkey; **MCSNV**, Museo Civico di Storia Natuale di Verona, Italy; **NHMG**, The Natural History Museum of Geneva, Switzerland; **AZM**, Alaşehir Zoological Museum, Manisa, Turkey; **ZMMU**, Zoological Museum, Moscow Lomonosov State University, Russia.

## Taxon treatments

### Harpactea alanyana sp. n.

urn:lsid:zoobank.org:act:F0D68FCC-5A3E-40B6-8F0D-A898C03D6D30

#### Materials

- **Type status:** Holotype. **Occurrence:** recordedBy: R.S. Özkütük; sex: 1 male; lifeStage: adult; preparations: whole animal (ETOH); disposition: in collection; **Taxon:** scientificName: Harpactea alanyana; class: Arachnida; order: Araneae; family: Dysderidae; genus: Harpactea; specificEpithet: alanyana; nomenclaturalCode: ICZN; **Location:** continent: Asia; country: Turkey; countryCode: TR; stateProvince: Mediterranean; county: Antalya; municipality: Alanya; locality: Taşatan Plateau; verbatimLatitude: 36°38'37.3500"; verbatimLongitude: 032°04'42.0900"; verbatimCoordinateSystem: degrees minutes seconds; **Event:** samplingProtocol: sifter; eventDate: 24 April 2011; habitat: pine forest; **Record Level:** institutionCode: AUZM; basisOfRecord: PreservedSpecimen
- **Type status:** Paratype. **Occurrence:** recordedBy: M. Elverici; sex: 1 male, 1 female; lifeStage: adult; preparations: whole animal (ETOH); disposition: in collection; **Taxon:** scientificName: Harpactea alanyana; class: Arachnida; order: Araneae; family: Dysderidae; genus: Harpactea; specificEpithet: alanyana; nomenclaturalCode: ICZN; **Location:** continent: Asia; country: Turkey; countryCode: TR; stateProvince: Mediterranean; county: Antalya; municipality: Alanya; locality: Asmaca Village; verbatimLatitude: 36°36'32.3000"; verbatimLongitude: 032°03'12.4000"; verbatimCoordinateSystem: degrees minutes seconds; **Event:** samplingProtocol: sifter; eventDate: 3 January 2013; habitat: pine forest; **Record Level:** institutionCode: NHMG; basisOfRecord: PreservedSpecimen
- **Type status:** Paratype. **Occurrence:** recordedBy: M. Elverici; sex: 2 female; lifeStage: adult; preparations: whole animal (ETOH); disposition: in collection; **Taxon:** scientificName: Harpactea alanyana; class: Arachnida; order: Araneae; family: Dysderidae; genus: Harpactea; specificEpithet: alanyana; nomenclaturalCode: ICZN; **Location:** continent: Asia; country: Turkey; countryCode: TR; stateProvince: Mediterranean; county: Antalya; municipality: Alanya; locality: Asmaca Village; verbatimLatitude: 36°36'32.3000"; verbatimLongitude: 032°03'12.4000"; verbatimCoordinateSystem: degrees minutes seconds; **Event:** samplingProtocol: sifter; eventDate: 3 January 2013; habitat: pine forest; **Record Level:** institutionCode: AUZM; basisOfRecord: PreservedSpecimen
- **Type status:** Paratype. **Occurrence:** recordedBy: K.B. Kunt; sex: 1 male, 1 female; lifeStage: adult; preparations: whole animal (ETOH); disposition: in collection; **Taxon:** scientificName: Harpactea alanyana; class: Arachnida; order: Araneae; family: Dysderidae; genus: Harpactea; specificEpithet: alanyana; nomenclaturalCode: ICZN; **Location:** continent: Asia; country: Turkey; countryCode: TR; stateProvince: Mediterranean; county: Antalya; municipality: Alanya; locality: Avsallar Town; verbatimLatitude: 36°38'21.5000"; verbatimLongitude: 031°45'24.9000"; verbatimCoordinateSystem: degrees minutes seconds; **Event:** samplingProtocol: sifter; eventDate: 6 January 2013; habitat: pine forest; **Record Level:** institutionCode: ZMMU; basisOfRecord: PreservedSpecimen
- **Type status:** Paratype. **Occurrence:** recordedBy: K.B. Kunt; sex: 1 male, 1 female; lifeStage: adult; preparations: whole animal (ETOH); disposition: in collection; **Taxon:** scientificName: Harpactea alanyana; class: Arachnida; order: Araneae; family: Dysderidae; genus: Harpactea; specificEpithet: alanyana; nomenclaturalCode: ICZN; **Location:** continent: Asia; country: Turkey; countryCode: TR; stateProvince: Mediterranean; county: Antalya; municipality: Alanya; locality: Avsallar Town; verbatimLatitude: 36°38'21.5000"; verbatimLongitude: 031°45'24.9000"; verbatimCoordinateSystem: degrees minutes seconds; **Event:** samplingProtocol: sifter; eventDate: 6 January 2013; habitat: pine forest; **Record Level:** institutionCode: ZMMU; basisOfRecord: PreservedSpecimen
- **Type status:** Other material. **Taxon:** scientificName: *Harpactea osellai* Brignoli, 1978; namePublishedIn: Brignoli P.M. 1978. Ragni di Turchia V. Specie nuove o interessanti, cavernicole ed epigee, di varie famiglie (Araneae). Revue suisse de Zoologie. Vol.85. P.461-541.; class: Arachnida; order: Araneae; family: Dysderidae; genus: Harpactea; specificEpithet: osellai; taxonomicStatus: accepted; **Location:** country: Turkey; countryCode: TR; stateProvince: Amasya; locality: Borabay Lake; **Event:** eventDate: 4 June 1969; **Record Level:** institutionCode: MCSNV

#### Description

**Measurements [Holotype ♂ / Paratype ♀]: AL** 1.88 / 3.00; **CL** 1.70 / 2.28; **CWmax** 1.30 / 1.72; **CWmin** 0.63 / 0.92; **AMEd** 0.08 / 0.11; **PLEd** 0.07 / 0.09; **PMEd** 0.05 / 0.08; **ChF** 0.28 / 0.40; **ChG** 0.25 / 0.27; **ChL** 0.64 / 0.93. Leg measurements are given in (Table 1).

No apparent dimorphism between the sexes except in body sizes. Carapace hexagonal, reddish brown, dull and smooth. Carapace covered with very short, tiny and sparsely distributed setae. AME, PLE and PME closely grouped; AME separated (Fig. 1a, b, c, d). Sternum yellowish. Margins of sternum brownish. Labium, gnathocoxae and chelicerae brownish; darker than sternum. Retrolateral edges at the distal part of the gnathocoxae with dense greyish-light brown setae. Distal part of gnathocoxae retrolaterally sclerotized. Anterior surface of chelicera with blackish, dark brown tubercles, with one seta on each.

Cheliceral groove with four teeth; at retromargin, with a small tooth located at the base of the groove, and with a more developed second tooth a little above the second quarter. Both retromarginal teeth conical and tubercular. Promarginal teeth more strongly developed; the one closer to the base of the cheliceral groove larger and almost twice the size of the other (Fig. 1e, f). Abdomen cylindrical, yellowish; covered with tiny brownish hairs dorsally and ventrally (Fig. 1a).

Legs yellowish brown, covered with tiny brownish hairs on all surface. Leg formula IV, I, II, III. Tarsi with three claws. Paired claws toothed. Paired claws of leg I and II with 7 teeth; leg III and IV with 4 teeth. Scopula weak and in ventral position on the first distal half on 3rd and 4th tarsi and on 4th metatarsus; relatively strongly developed on 3rd metatarsus in ventral position at the first distal half. Coxae III with 1 prolateral spine; coxae IV with 1-2 prolateral spines. Patellae III with 1 spine dorsally. Further details on leg spination are given in (Table 2).

##### Palp

Bulbus almost oval, yellowish brown, with embolus and two apophyses. An apparent membranous part between the bulb and the distal appendages; embolus hook-shaped, black, almost homogenously sclerotized. Bent anteriorly following the same course as apophysis_b_. Apophysis_a_ L-shaped, apically well sclerotized, short and strong. Apophysisb separated from the membranous part of the bulbus with a wide angle from the base and orientates anteriorly. Apically blunt, conical and in the shape of a triangular apophysis in retrolateral view (Fig. 2).

##### Vulva

Distal expansion of spermatheca tenuously developed between distal crest and rod-shaped part of the anterior spermatheca. Nevertheless, it is more sclerotized at the surface compared to other parts of the anterior spermatheca. Distal crest gradually tapers through the tip. Rod-shaped part of the anterior spermatheca almost one and a half times the length of the distal crest. Basal transverse part of the anterior spermatheca widens through laterals and narrows through the tips, in the shape of open wings. A ring-shaped strongly sclerotized structure is apparent at the juncture of the rod-shaped part of the anterior spermatheca and the basal transverse part of the anterior spermatheca. Transverse bar in the shape of a lip. Posterior diverticulum prominent, and in the form of a broad membranous sac (Fig. 3).

#### Diagnosis

*Harpactea alanyana* sp. n. can be easily distinguished from all known species of *Harpactea* by the unique structures of the male and female copulatory organs. The male palp of the new species is similar to that of *H. osellai*. However, the two species can be easily separated by the shape of the bulb; by having a less curled embolus compared to *H. osellai* and by the shape of the distal apophysis (Fig. 4a, b). Additionally, the two species also differ in their body sizes and by the morphology of the female vulvae (Fig. 4c).

#### Etymology

The specific name refers to the type locality (Alanya District, Antalya Province, Turkey).

#### Distribution

*Harpactea alanyana* sp. n. is currently known only from the type locality and its vicinity.

#### Ecology

All specimens were collected from slopes and ridges lying parallel to the coastline, from sea level up to a maximum of around 1200 m on peaks of the Taurides Mountain range, by sifting tree litter of mixed forest with the following species *Pinus nigra*, *Quercus coccifera*, *Arbutus andrachne*, *Ceratonia siliqua*, etc. Adult males are known to be active from late autumn until the middle of spring, while females are only known from the winter.

#### Taxon discussion

Brignoli (1978a) placed *H. osellai* under *hombergi* group, *babori* subgroup. A wide or flat embolus and a conspicuous Ap_a_ are characteristics of the *babori* subgroup.

Deeleman-Reinhold (1993) classified *Harpactea* under four major groups by considering structure of male and female reproductive organs, spination on legs and partly biogeographic distributions of the species. Among these groups, "D. group *rubicunda*" is characterized by the structure of vulva with a broad and membranous posterior diverticulum on female, and on male generally by the structure of bulb being globular and embolus and conductor (Ap_a_) being massive. Besides, coxae IV and patellae III can have 1 or more spines.

*H. alanyana*;

**1.** Based on the oval bulbus, the massive embolus and apophysis_a_,

**2.** the presence of spines on the patellae and coxae,

"*H. alanyana*" belongs to "D. group *rubicunda*" by the characters stated above.

#### Notes

Brignoli (1978a) indicated in the label information that the holotype of *H. osellai* had been preserved in “The Natural History Museum of Geneva” and the paratype in the “Civic Museum of Natural History of Verona”. We incidentally learned that it was the opposite, which means that the male holotype of *H. osellai* is currently held in the “Civic Museum of Natural History of Verona” and the female paratype is found in “The Natural History Museum of Geneva”.

## Supplementary Material

XML Treatment for Harpactea alanyana

## Figures and Tables

**Figure 1a.**
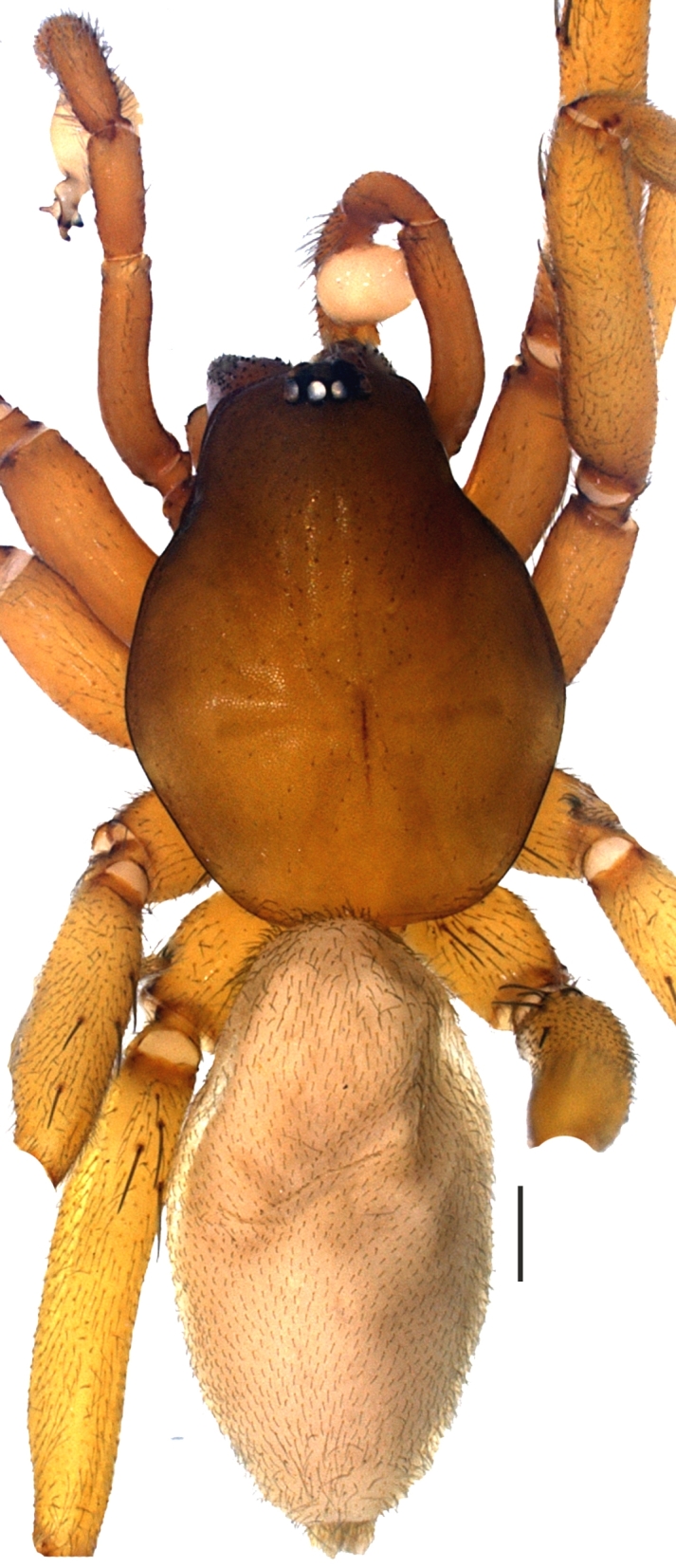
Habitus of holotype male

**Figure 1b.**
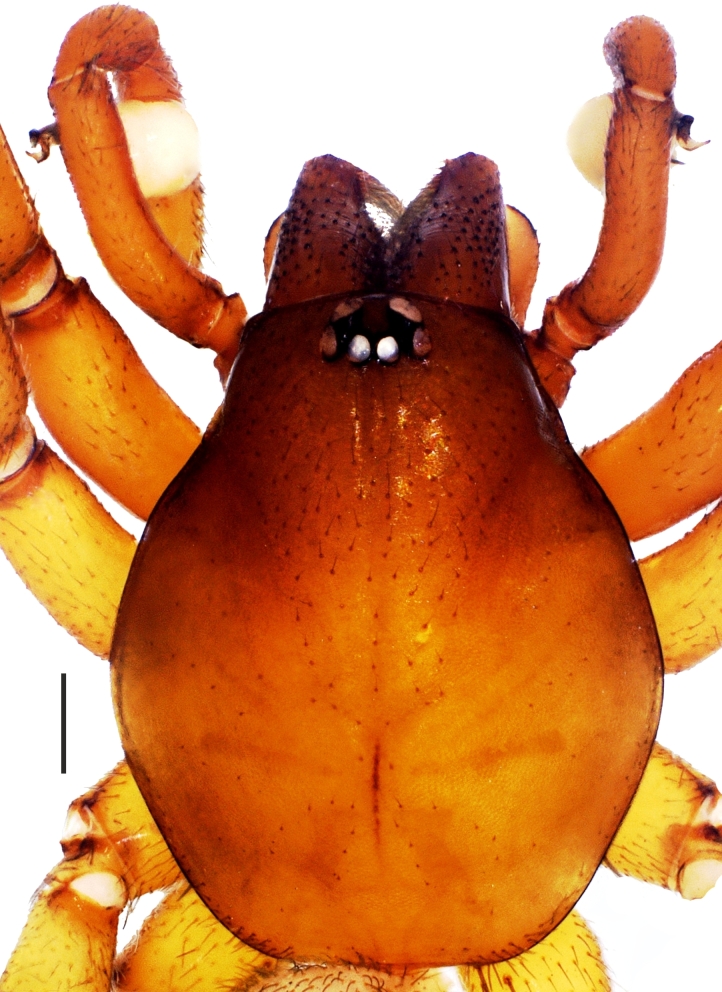
Carapace, male

**Figure 1c.**
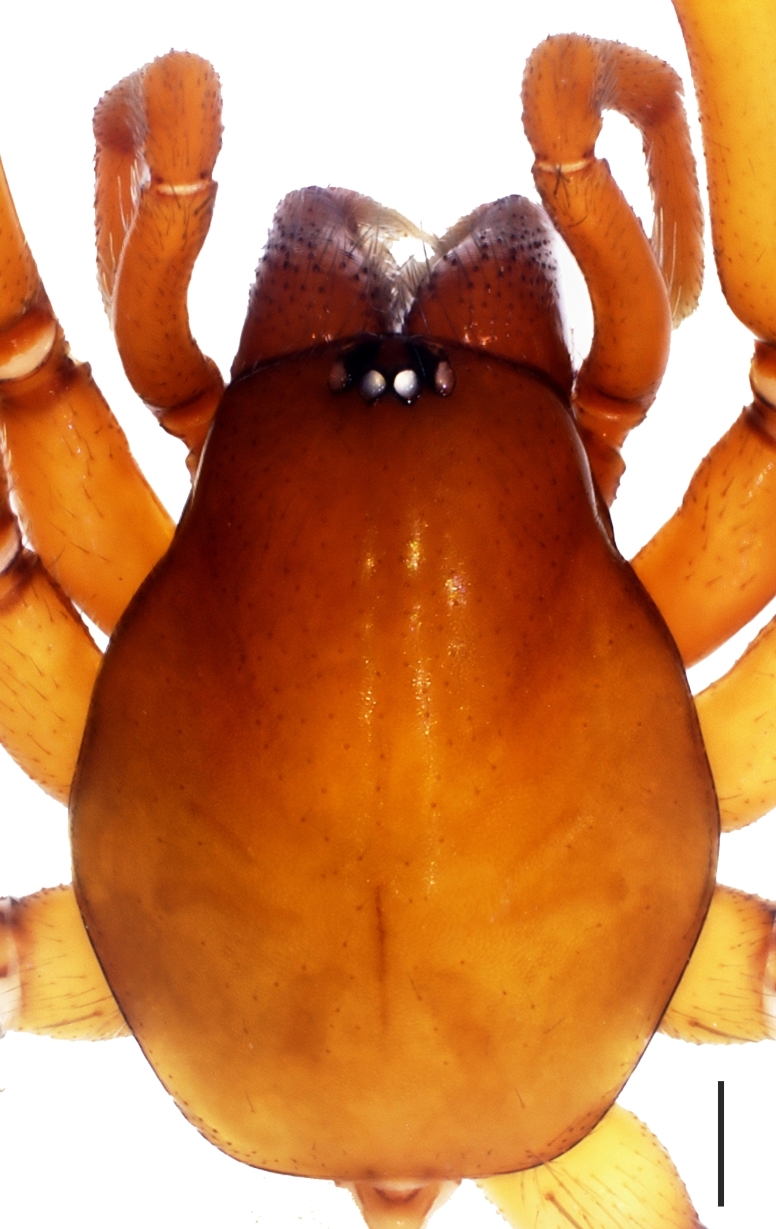
Ditto, female

**Figure 1d.**
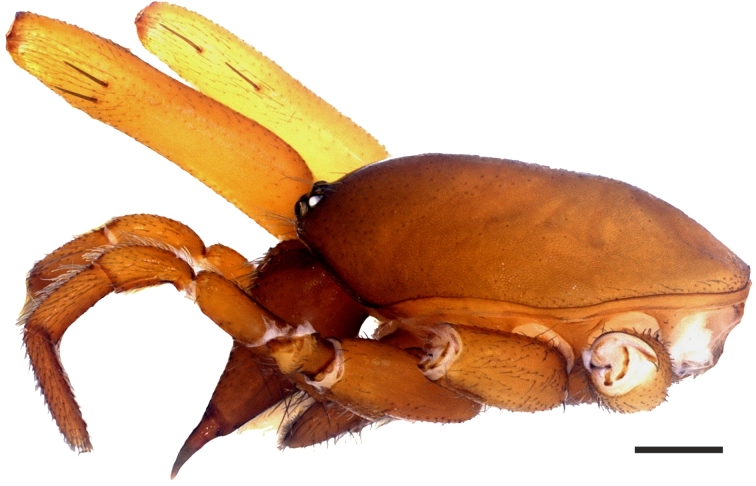
Ditto, lateral view

**Figure 1e.**
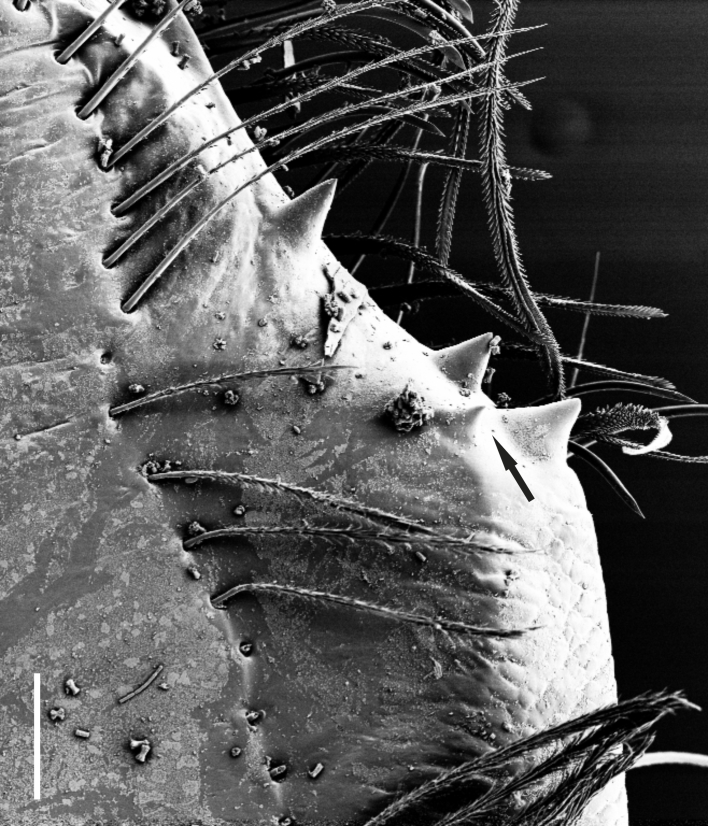
Cheliceral teeth (arrow indicates position of retromarginal small tooth)

**Figure 1f.**
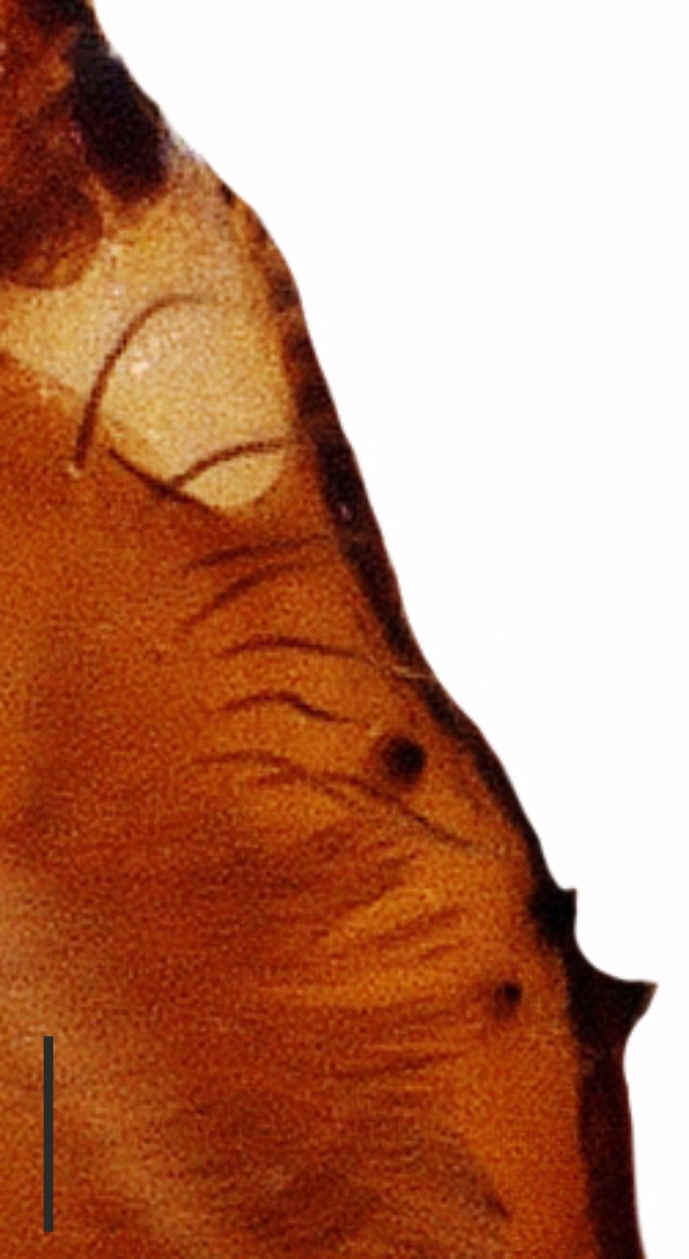
Ditto

**Figure 2a.**
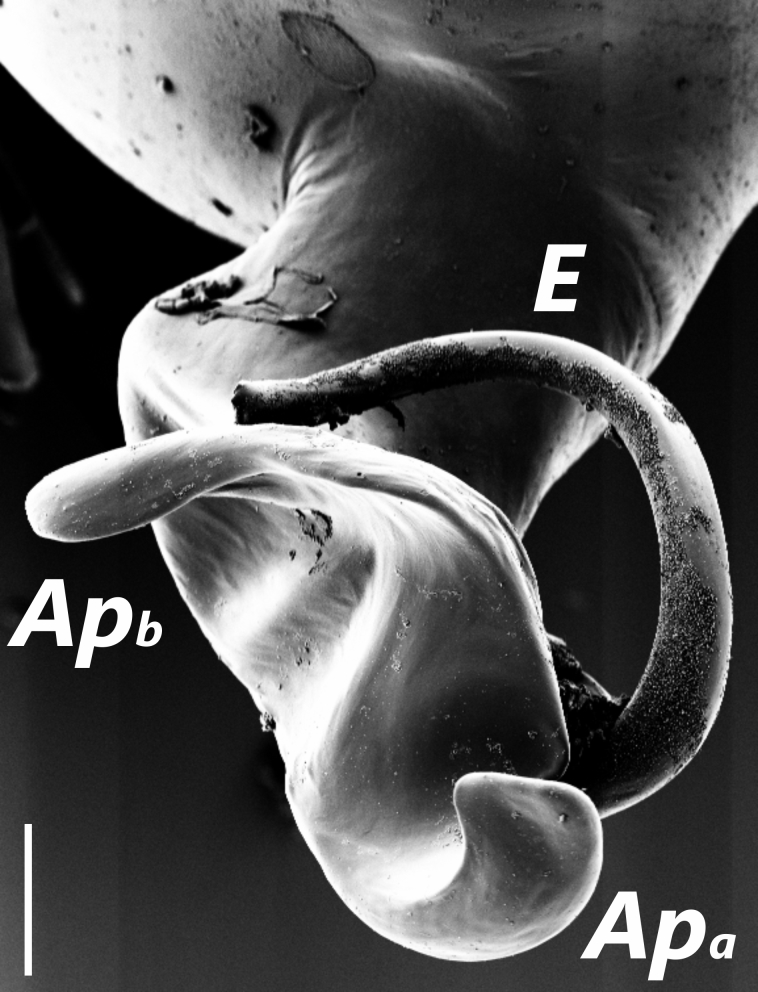
Distal appendages of male bulb, nearly posterior view. Abbreviations: ***Ap_a_***, apophysis_a_; ***Ap_b_***, apophysis_b_; ***E***, Embolus

**Figure 2b.**
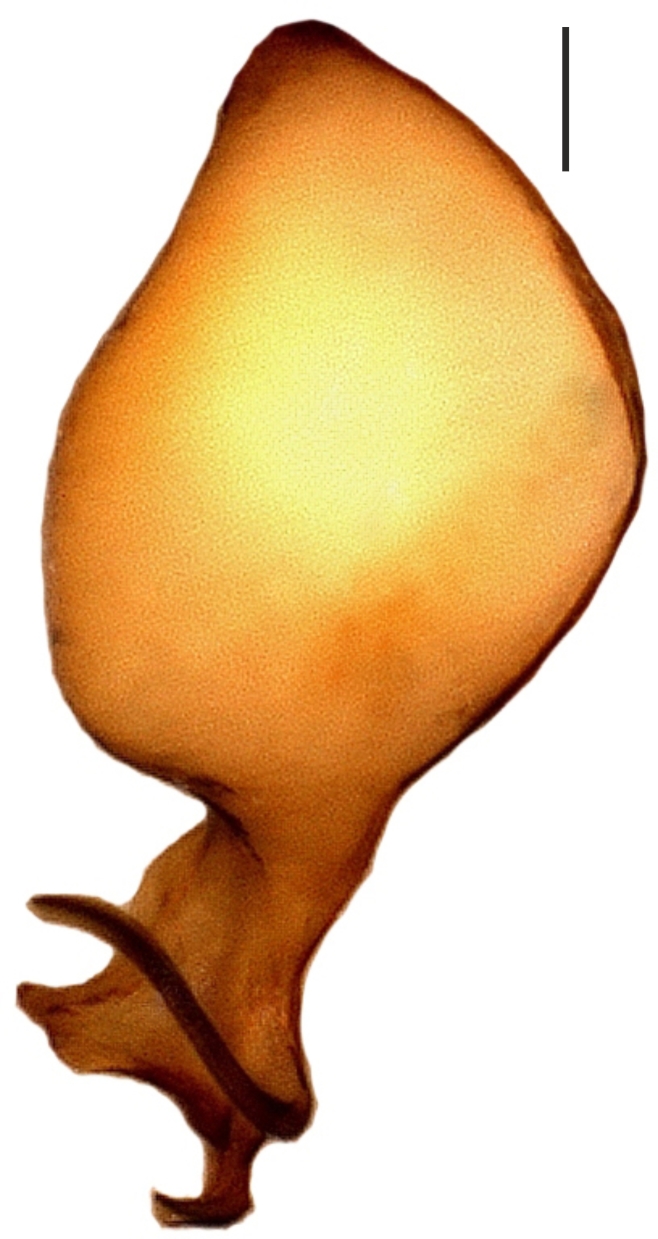
Male palp, prolateral view

**Figure 2c.**
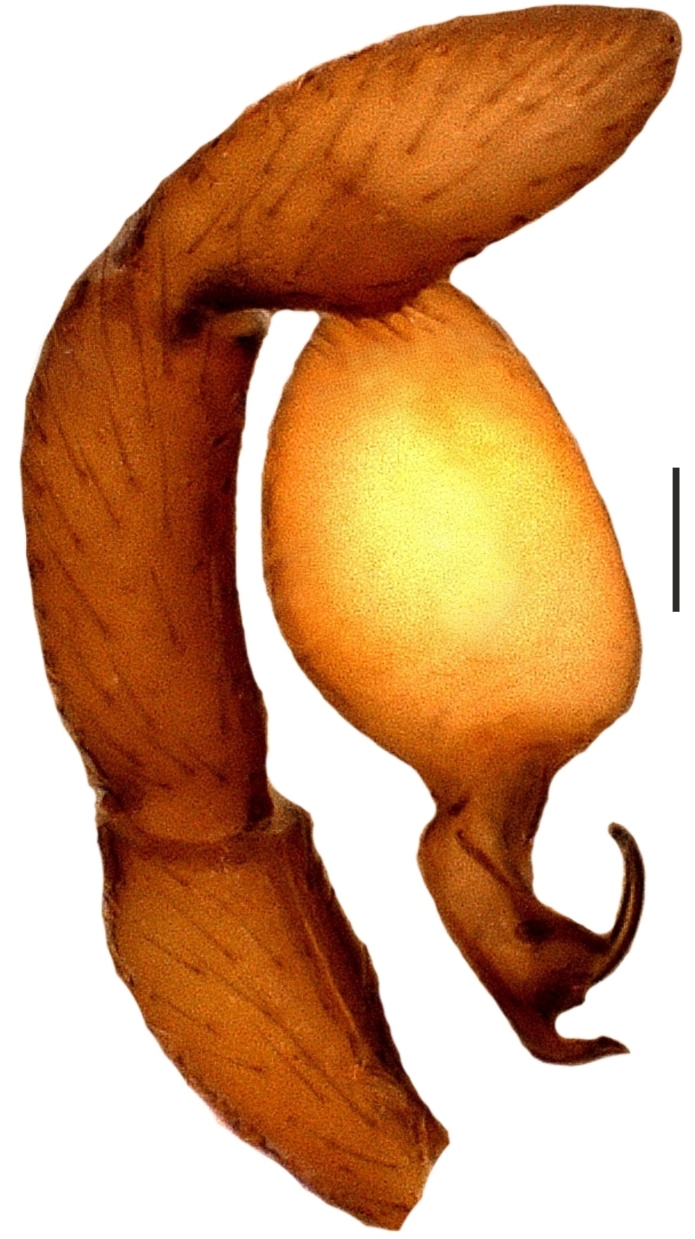
Ditto, retrolateral view

**Figure 2d.**
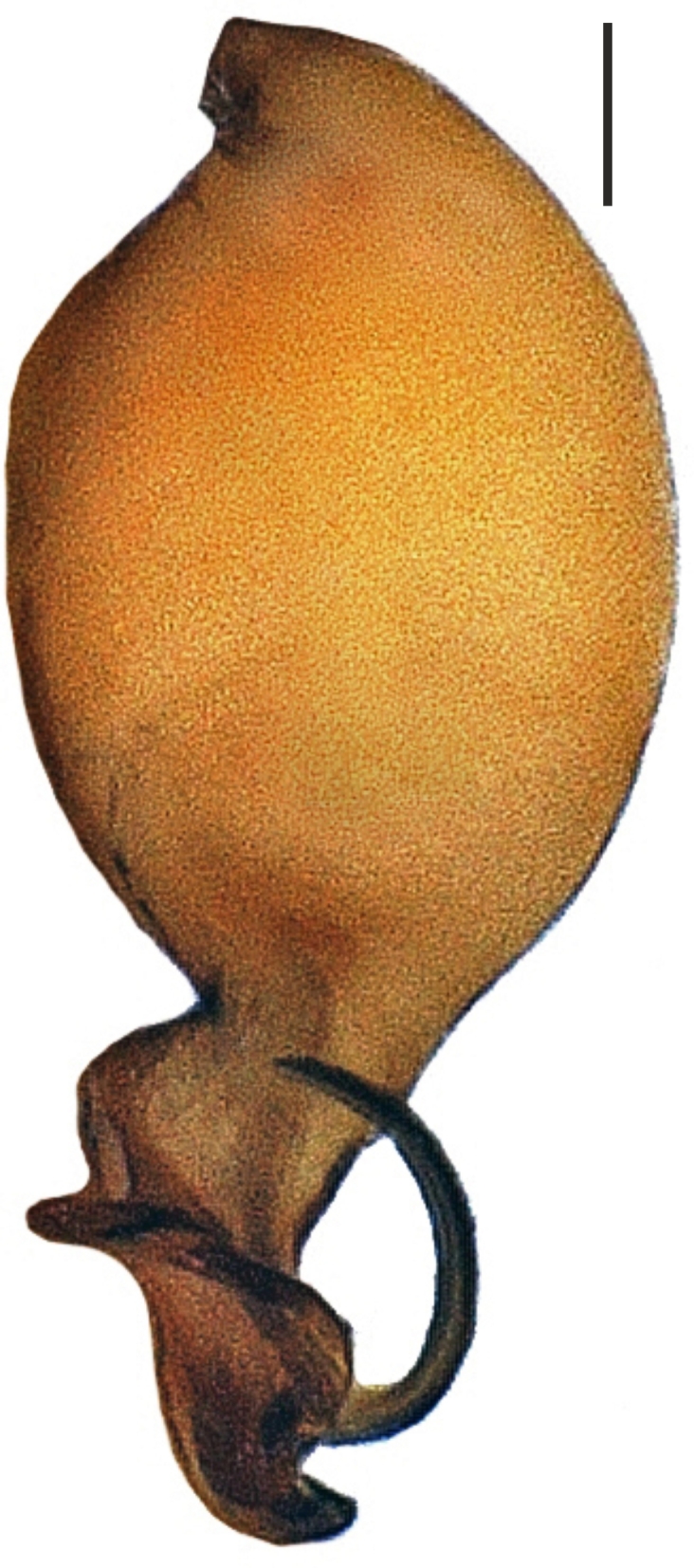
Ditto, nearly retrolateral view

**Figure 3a.**
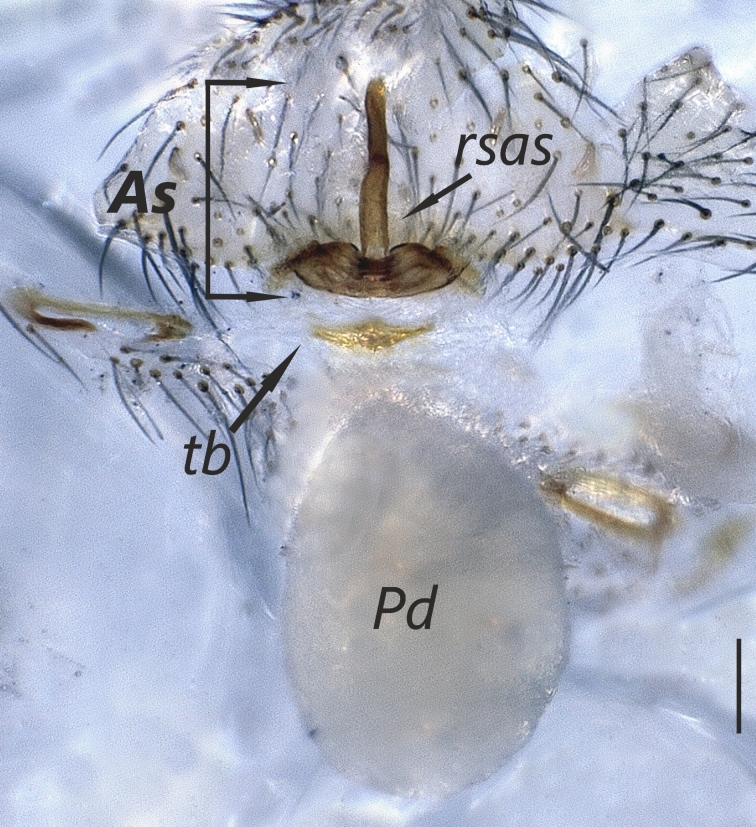
Vulva, dorsal view

**Figure 3b.**
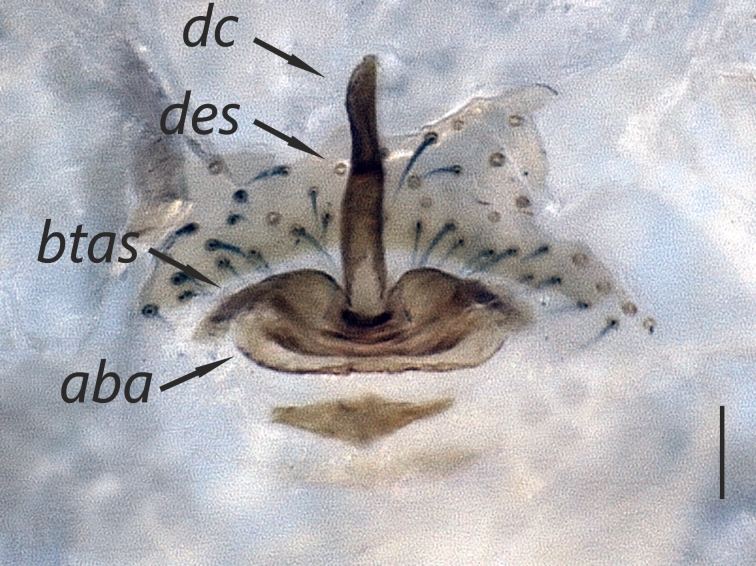
Ditto

**Figure 3c.**
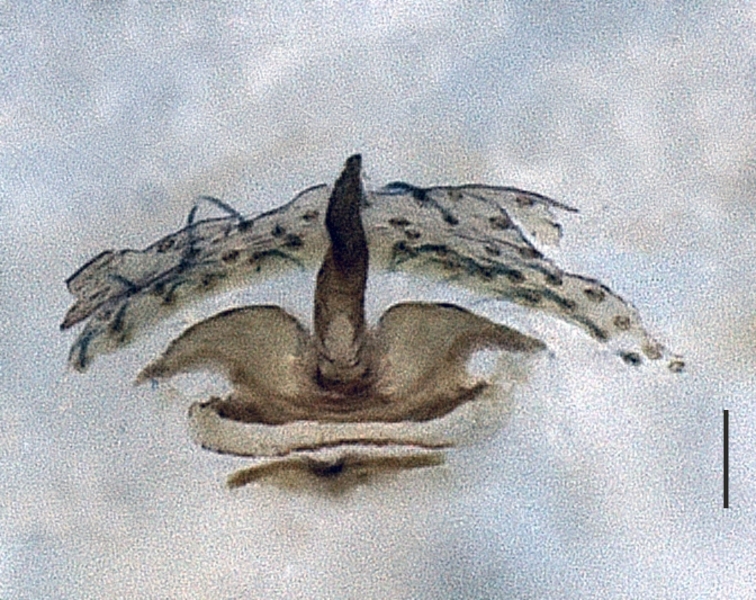
Ditto

**Figure 3d.**
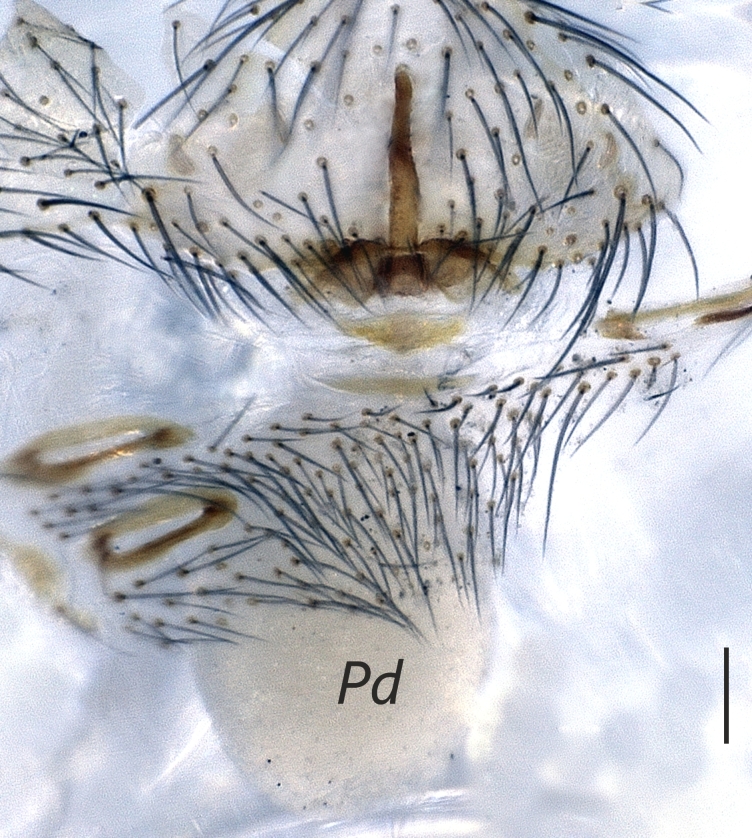
Ditto, ventral view

**Figure 4a.**
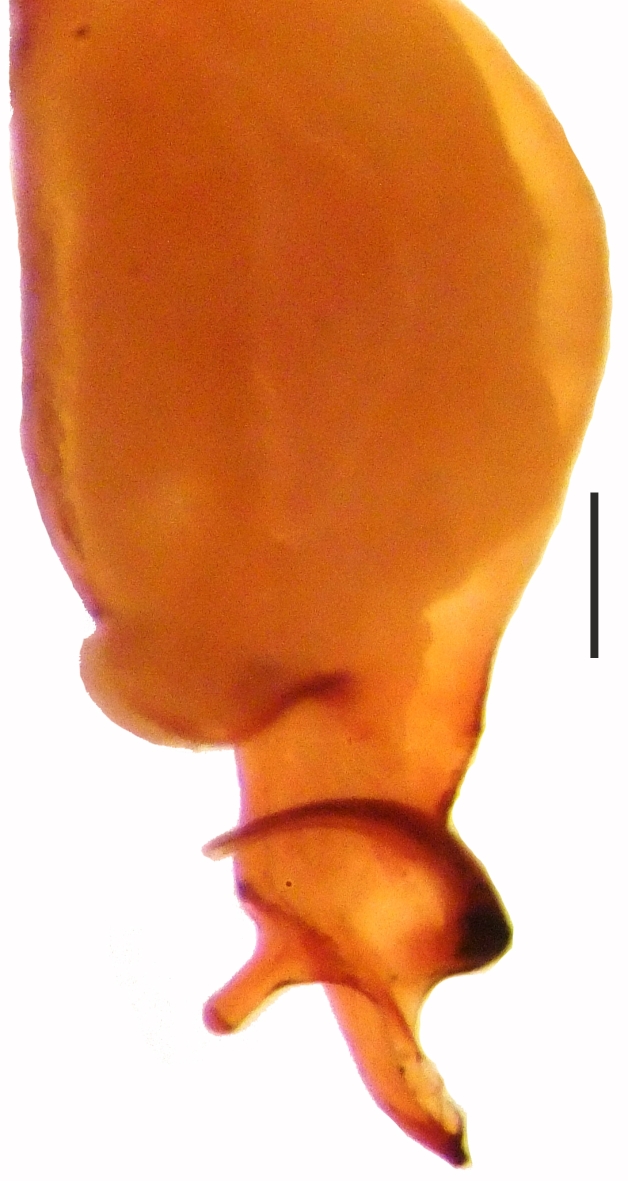
Male palp, prolateral view

**Figure 4b.**
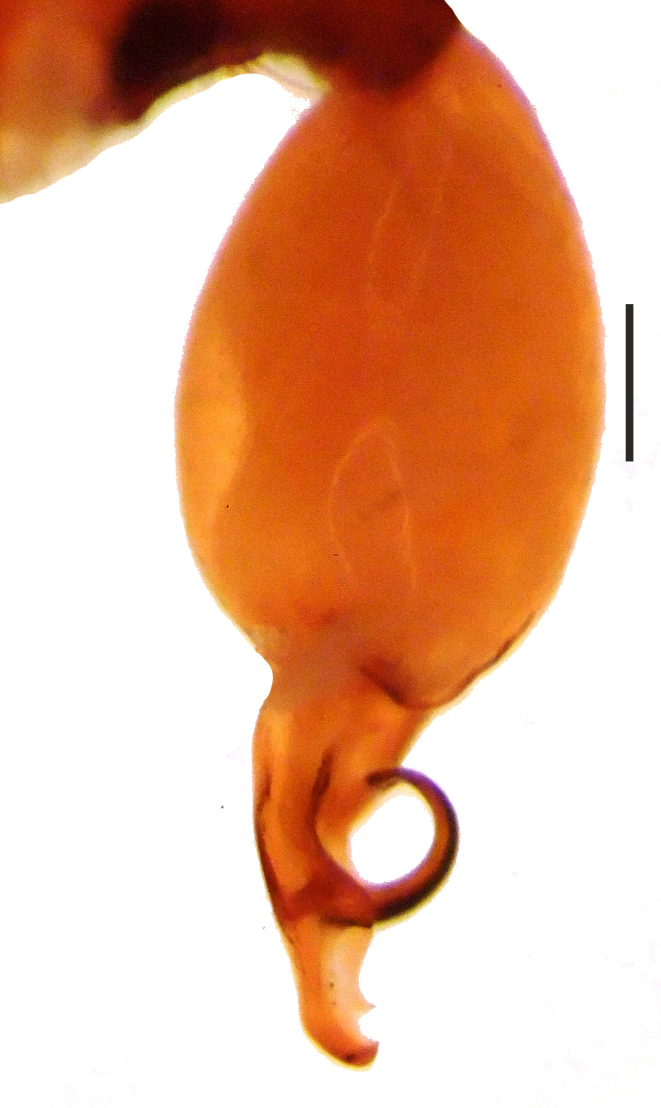
Ditto, retrolateral view

**Figure 4c.**
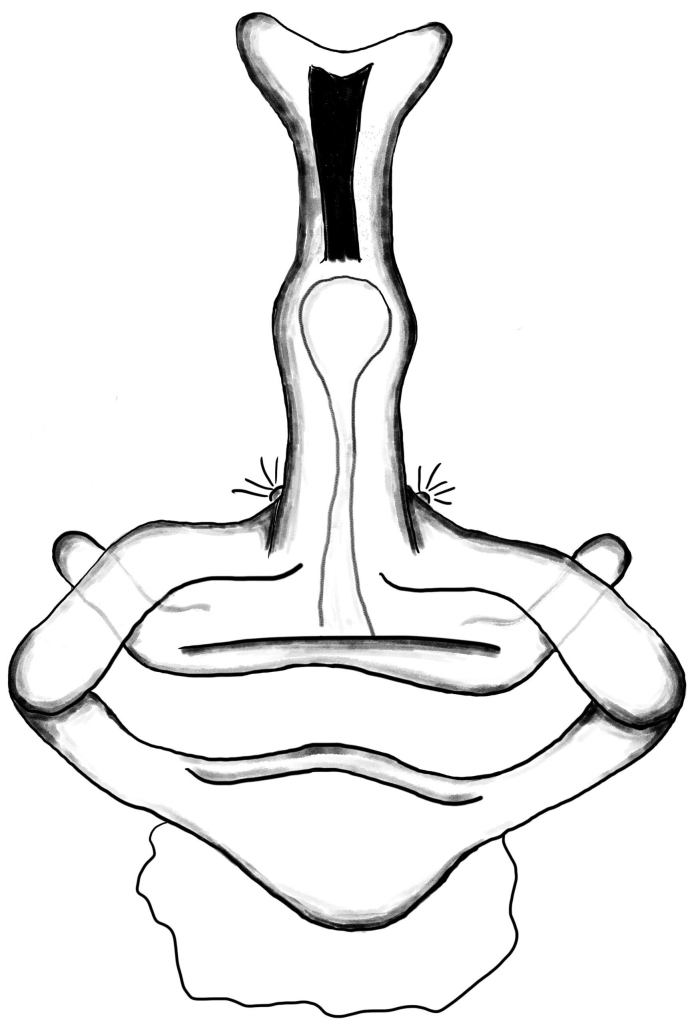
Vulva, dorsal view.

**Table 1.** Leg measurements of *Harpactea alanyana* sp. n. (Holotype **♂** / Paratype **♀**)

**Leg**	**Fe**	**Pa**	**Ti**	**Me**	**Ta**	**Total**
**I**	1.48 / 1.88	0.83 / 1.14	1.18 / 1.55	1.00 / 1.35	0.40 / 0.45	4.89 / 6.37
**II**	1.35 / 1.75	0.75 / 1.13	1.13 / 1.45	1.10 / 1.38	0.38 / 0.38	4.71 / 6.09
**III**	1.07 / 1.45	0.57 / 0.73	0.83 / 0.78	1.10 / 1.38	0.34 / 0.48	3.91 / 4.82
**IV**	1.56 / 2.00	0.75 / 0.98	1.38 / 1.53	1.41 / 1.78	0.50 / 0.53	5.60 / 6.82

**Table 2.** Leg spination of *Harpactea alanyana* sp. n.

**♂**	**Leg I**	**Leg II**	**Leg III**	**Leg IV**
**C**	0	0	1 Pl	1 Pl
**Fe**	2 Pl	1, 1 Pl	1, 1 D 1, 1, 1 Rl	1, 2 D
**Pa**	0	0	1 D	0
**Ti**	0	0	1, 1 Pl 1, 1, 1 Rl 1, 1, 2 V	1, 1, 1 Pl 1, 1, 1 Rl 1, 1, 2 V
**Me**	0	0	1, 1 Pl 1, 1, 1 Rl 2, 1, 2 V	1, 1, 1 Pl 1, 1, 1, 1 Rl 1, 1, 2 V
**♀**				
**C**	0	0	1 Pl	1-2 Pl
**Fe**	2 Pl	1, 1 Pl	1, 1 D 1, 1 Rl	1, 1 Pl 1, 1 D
**Pa**	0	0	1 D	0
**Ti**	0	0	1, 1 Pl 1, 1, 1 Rl 1, 1, 2 V	1, 1, 1 Pl 1, 1, 1 Rl 1, 1, 2 V
**Me**	0	0	1, 1 Pl 1, 1, 1 Rl 2, 1, 2 V	1, 1, 1 Pl 1, 1, 1, 1 Rl 1, 1, 2 V
